# Innovative Uses for Syndromic Surveillance

**DOI:** 10.3201/eid1604.090688

**Published:** 2010-04

**Authors:** Erin K. O’Connell, Guoyan Zhang, Fermin Leguen, Anthoni Llau, Edhelene Rico

**Affiliations:** Miami–Dade County Health Department, Miami, Florida, USA

**Keywords:** Syndromic surveillance, communicable disease, queries, clustering, outbreaks, infectious disease, bacteria, viruses, parasites, dispatch

## Abstract

To determine if expanded queries can be used to identify specific reportable diseases/conditions not detected by using automated syndrome categories, we developed new categories to use with the Electronic Surveillance System for the Early Notification of Community Based Epidemics. Results suggest innovative queries can enhance clinicians’ compliance with reportable disease requirements.

Surveillance and control of communicable diseases are critical for the health status of a community. Traditional passive surveillance refers to health authorities’ receipt of reports of diseases or conditions submitted by physicians, laboratories, and other healthcare providers as required by public health legislation. However, reportable diseases are often underreported to health departments (*1*,*2*). Syndromic surveillance has been defined as “an investigational approach where health department staff, assisted by automated data acquisition and generation of statistical alerts, monitor disease indicators in real-time or near real-time to detect outbreaks of disease earlier than would otherwise be possible with traditional surveillance” (*3*). Since 2005, the Miami–Dade County Health Department has used the Electronic Surveillance System for the Early Notification of Community Based Epidemics (ESSENCE) as part of its comprehensive syndromic surveillance system. The system categorizes chief complaints into 11 syndromes: botulism-like, exposure, fever, gastrointestinal illness, hemorrhagic illness, influenza-like illness, injury, neurologic, rash, respiratory, and shock-coma. Of the county’s 23 acute-care hospitals, the 17 largest, which account for 90% of the county’s emergency department visits, participate in ESSENCE. Staff epidemiologists rotate duties and dedicate 2 hours a day, including weekend, to syndromic surveillance activities. Monday through Friday daily reports are sent to community partners.

Syndromic surveillance was primarily designed to detect disease outbreaks and unusual public health events earlier than could be detected by traditional public health surveillance methods. However, if an outbreak or cluster of illness is too small, the method used currently for syndromic surveillance cannot trigger a statistical alert. We wanted to ascertain the value of syndromic surveillance in improving regular communicable disease surveillance and reporting. The possibility of using ESSENCE to detect specific diseases emerged when varicella (chickenpox) became a newly reportable disease in Florida in 2006; few cases were being reported despite the fact that guidelines had been mailed to all healthcare practitioners. We used a query in ESSENCE to search for “chicken pox or varicella” in the chief complaint field and contacted the hospital Infection Control Practitioners to verify if identified events could be confirmed as reportable conditions. After we did preassessment of the underreporting of chickenpox in 2007 (*4*), three additional query categories for daily investigation were created in ESSENCE.

## The Study

The following category queries were performed during March–December 2008: 1) Severe or time-sensitive diseases/conditions: anthrax, botulism, smallpox, and meningitis; 2) outbreaks not detected by regular alerts in ESSENCE: diarrhea, vomiting, and food poisoning with spatial-temporal clustering; and 3) other reportable diseases/conditions, consisting of varicella (chickenpox), carbon monoxide poisoning, ciguatera, cryptosporidiosis, cyclosporiasis, dengue fever, encephalitis, hepatitis, malaria, measles, mercury poisoning, mumps, pertussis, salmonellosis, shigellosis, and rabies.

When descriptions of the diseases/conditions were found in the chief complaint field, staff contacted the hospital’s Infection Control Practitioner. If the disease was confirmed, further investigation was performed. Potential outbreaks were detected from clustering by age, gender, race/ethnicity, resident ZIP code, hospital, time of visit, and chief complaint.

A total of 740,320 emergency department visits (mean 2,419 visits/day) were monitored in ESSENCE during the study period; 1,813 (0.25%) of those had information leading to 1 of the queried reportable diseases in the chief complaint (mean 5.9 visits/day). After further investigation, we found 58.0% (1,052/1,813) of these additional queries were relevant after excluding unrelated terms, such as “malabsorption” instead of “malaria” or “chicken bone in the throat” instead of “chicken pox.”

On August 31, 2008, the newly designed query for severe or time-sensitive diseases or conditions detected a group of 5 women who had arrived at the same hospital within a 2-hour period with a chief report of either meningococcemia or exposure to meningococcemia (Table 1). This cluster of potentially epidemiologically linked patients did not initiate an automated alert in a syndrome in ESSENCE, and the hospital did not report it by phone immediately. Therefore, without the query, the health department would not have been aware of these patients in a timely manner. The findings from the query enabled health department staff to give postexposure prophylaxis to 36 persons identified as close contacts.

**Table 1:** Emergency department visits with meningococcemia in the chief complaint, Miami–Dade County, Florida, USA, August 31, 2008*

Time of visit	Patient age, y	Chief complaint
9:48 pm	3	Exposure to meningococcemia
9:47 pm	8	Exposure to meningococcemia
9:45 pm	11	Exposure to meningococcemia
11:50 pm	9	Exposure to meningococcemia
11:58 pm	10	Meningococcemia

*All 5 patients were girls who visited the same hospital and came from the same ZIP code area.

Gastrointestinal outbreaks are an example of outbreaks not detected by regular alerts in ESSENCE that were often detected through the use of the specialized query. One outbreak identified was among persons staying in the same homeless shelter (Table 2), another was among residents at an assisted living facility, and a third was among a group of persons who visited a restaurant. After the outbreaks were confirmed with the Infection Control Practitioner, recommendations for infection control and prevention were made to each facility.

**Table 2:** Disease/conditions found in the chief complaint field from specialized query, Miami–Dade County, Florida, USA, March–December 2008

Disease/condition	No. (%) cases
Food poisoning	262 (24.9)
Postexposure prophylaxis for rabies	251 (23.9)
Meningitis	224 (21.3)
Hepatitis	162 (15.4)
Chicken pox	80 (7.6)
Carbon monoxide poisoning	31 (3.0)
Salmonellosis	13 (1.2)
Malaria	9 (0.9)
Pertussis	8 (0.8)
Mumps	5 (0.5)
Other*	5 (0.5)
Total	1,052 (100)

*Ciguatera, anthrax, dengue fever, measles.

The most common terms found under other reportable diseases or conditions were meningitis, hepatitis, chicken pox, and postexposure prophylaxis for rabies (Table 2). These 4 conditions accounted for 68.2% (717/1,052) of the queried chief complaints. When we contacted providers with regard to query findings, it was apparent that some providers were not familiar with reporting requirements for chicken pox and animal bites requiring postexposure prophylaxis for potential rabies exposure.

## Conclusions

Results suggest ESSENCE can enhance healthcare practitioners’ compliance with reportable disease requirements for individual diseases and potential outbreaks. Results demonstrated how expanded queries can detect potential outbreaks or diseases not found in automated syndrome categories. Without the specialized queries, we would have missed the opportunity to implement proper disease control measures required for these events.

Indiana State Health Department investigators found that certain keywords such as “exposure” and “meningitis” may uncover trends previously undetected and they continue to explore similar data-mining techniques (*5*). Syndromic surveillance systems use statistical algorithms to alert users when the number of reports for a syndrome exceeds the norm (*6*). Current spatial–temporal algorithms are used to detect large-scale outbreaks over a certain extended period (*7*,*8*). However, this method has not been successful for detecting many small clusters of patients with similar characteristics visiting the emergency department from the same home ZIP code, hospital, or within a short period, such as a few minutes. By contacting the Infection Control Practitioner when reportable disease names are found in the chief complaint field, the health department has developed a stronger relationship with hospitals.

One of the limitations of the study was that even when queries were performed with parsers, there were often misspellings, typographical errors, and abbreviations that can lead to a failure to capture all possible events (*9*). Because the level of investigation can vary from making a phone call to a participating hospital to dispatching a team to interview patients, the cost of time spent on each disease may need to be weighed before initiating action (*10*). Replication of this study depends on a health department’s capabilities to contact the hospital for follow-up. Future research will examine the information gathered from this new project, and we expect that better disease reporting compliance will result from this innovative use of syndromic surveillance.
